# Expanded Newborn Screening Using Tandem Mass Spectrometry: Seven Years of Experience in Eastern Sicily

**DOI:** 10.3390/ijns4020012

**Published:** 2018-04-05

**Authors:** MariaAnna Messina, Concetta Meli, Federica Raudino, Annarita Pittalá, Alessia Arena, Rita Barone, Fortunata Giuffrida, Riccardo Iacobacci, Vera Muccilli, Giovanni Sorge, Agata Fiumara

**Affiliations:** 1 Referral Center for Inherited Metabolic Diseases, Pediatric Clinical, AOU Policlinico-VE, Via Santa Sofia 78, 95123 Catania, Italy; 2 Child Neurology and Psichiatry, AOU Policlinico-VE, Via Santa Sofia 78, 95123 Catania, Italy; 3 Chemistry Department, Uiversity of Catania, Viale Andrea Doria 5, 95123 Catania, Italy

**Keywords:** newborn screening, mass spectrometry, inherited diseases

## Abstract

The expanded newborn screening for selected inborn errors of metabolism (IEM) in Sicily was introduced in 2007 by a Regional project entitled “Early detection of congenital metabolic diseases: expanded neonatal screening”. It established two newborn screening laboratories, for Western and Eastern Sicily, which started their activity in 2011. Here we present the results of expanded screening (excluding phenylketonuria (PKU)) of the Eastern laboratory from January 2011 to December 2017. Our data highlight the importance of the expanded newborn screening as a basic health program to avoid the underestimation of rare diseases and the need of further investigations even when there are no textbook alterations of the metabolic profiles. We performed our analysis on dried blood spot by tandem mass spectrometry, according to Italian guidelines. A total of 196 samples from 60,408 newborns gave positive screening results (recall rate 0.32%) while 12 babies were true positive, including 2 newborns whose mothers resulted in being affected by a metabolic disease. The overall frequency of IEM found in the screening panel was 1:6041 (mothers excluded) or 1:5034 (mothers included). The introduction of MS/MS technology in Sicily has significantly increased the detection of inherited metabolic disorders, including those not previously covered, with a predictable improved outcome for several disorders.

## 1. Introduction

Newborn screenings (NBS) provide an opportunity to identify several inherited rare metabolic disorders in pre-symptomatic infants, allowing to prevent or mitigate adverse outcomes associated with many of these conditions. Screening is accomplished by the analysis of biomarkers in a micro-sample of blood, collected from newborns using a variety of analytic methodologies. The range of detectable disorders was significantly expanded by electrospray ionization tandem mass spectrometry (ESI-MS/MS) technology, which enabled the simultaneous identification and quantification of several markers of fatty acid oxidation and amino acid disorders [1,2,3,4,5].

Even though for many of these disorders, treatment is not completely curative, new strategies are available or foreseen. Moreover, an early diagnosis relieves families of a severely ill child from long-lasting and difficult diagnostic procedures and allows appropriate genetic counseling [6].

### Newborn Screening in Sicily

In many countries, newborn screening is performed as standard practice on all babies, while in others, it is not feasible, or it is only available to a subset of neonates. Thus, the panel of disorders screened for, the procedures, and the cohort of newborns screened varies substantially in the different parts of the world [7,8]. In Sicily, the Expanded Newborn Screening Program was introduced and financed by a Regional Project, inside the Healthy National Plan of the Ministry of Health. It was initiated in 2011 and performed by two different Laboratories, according to the different geographical areas: Western and Eastern Sicily. However, during the first seven years, due to having to wait for screening program institutionalization by the National Healthcare System and due to the limited financial resources, the two Centers operated on a smaller geographical area than expected. Therefore, our laboratory, belonging to the Regional Referral Center for Inborn Errors of Metabolism, Pediatric Clinic, Polyclinic of Catania, has performed the Newborn Screening Program for the district of Catania. In this study, we report our seven years’ experience concerning MS/MS expanded newborn screening of 60,408 newborns in the district of Catania.

## 2. Materials and Methods

Dried blood spot (DBS) samples were collected from infants born in the district of Catania from January 2011 to December 2017. The Sicilian NBS program was based on voluntary participation, so parents’ consent was required. The study was approved by Regional Referral Center for Inborn Errors of Metabolism, Pediatric Clinic, Polyclinic of Catania on 3 November 2010. The total number of infants screened was 60,408. According to the suggested Italian Guidelines [9], blood samples were collected between 36 and 72 h into being born and a special protocol was applied to special conditions. Indeed, in cases of blood transfusion or after parenteral feeding, a second specimen collected after 7 days of life was required, while a second specimen collected after 15 days of life was required for preterm or low birthweight (<2400 g) babies; a third specimen collected after 30 days of life was required for very low birthweight babies (<1800 g). The blood samples were spotted on a Whatman 903TM filter paper (Eastern Business Forms, Grreenville, SC, USA), dried and sent, three times a week, by courier to our laboratory. Unsatisfactory specimens were rejected and re-sampling was done. The samples were stored in the same lab. Using an automatic puncher (Perkin-Elmer DBS Puncher, Perkin-Elmer, Turku, Finland), filter paper disks (3.2 mm in diameter) were punched out from the DBS into the wells of a microplate and 100 μL of a working solution (Neobase™ Non-derivatized MSMS kit, Perkin-Elmer) was added into each well. The plate was then shaken in the incubator/shaker (Wallac NCS incubator, Perkin-Elmer) at 45 °C for 45 min at a speed of 750 rpm. Then, 75 μL from the contents of each well was transferred to another microplate to avoid the presence of the paper disk during the injection in the mass spectrometer. We performed ESI-MS/MS analysis using a Waters Quattro Micro spectrometer equipped with a triple quadrupole. Each sample was injected by an autosampler (Waters 2777 C, Waters Corporations, Manchester, UK) and eluted by an HPLC pump (Waters 1525µ) at a flow rate of 0.16 mL/min, for 2 min. The MS parameters were set up as follows: capillary voltage 4 kV, source temperature 120 °C, desolvation temperature 350 °C, gas flow desolvation 640 L/h, and cone gas 50 L/h. In order to improve the sensitivity, the analytical measurements were performed in the multiple reaction monitoring mode (MRM). The stable isotope amino acids and acylcarnitines internal standards allowed the simultaneous determination of succinylacetone, 11 amino acids, free carnitine, and 30 different acylcarnitines. In order to monitor the performances of our assays, quality control (QC) was run with the same samples plate. The QC were provided by Perkin Elmer and consisted of whole human blood spotted onto Whatman 903 paper. The assigned concentration and standard deviation for each analyte were provided on a control label by Perkin Elmer. We set the upper and lower limits for the controls at ± 3 standard deviations of the assigned concentration. The list of the disease markers and the relative disorders detected in the MS/MS analyses were selected according to the Italian Guidelines panel.

The cut-off values used were initially set according to the literature. Subsequently, in order to established analyte reference levels, a healthy population consisting of 5000 subjects was considered. Data from a routine newborn screening of these 5000 specimens were determined. Then, the analyte concentrations corresponding to the 95th percentile were calculated. The obtained values were set up as cut-offs. The cut-off values are updated every 10,000 analyses.

When the results from a newborn sample exceeded the cut-off value, two different subsequent procedures were followed:

- In case of disorders at risk of metabolic decompensation during the neonatal period, a metabolic specialist immediately recalls the baby, and clinical examinations and confirmatory tests were performed.
- In case of all other disorders, the nursery was contacted to provide for a second DBS. If the repeated test showed a positive result, clinical examinations and confirmatory tests were performed.

In both cases, if the results from the confirmatory test were positive, the metabolic clinicians provide management, counseling, and follow-up.

## 3. Results

The adhesion of the maternities to the Sicilian NBS program took place gradually. Table 1 describes the number of screened babies in the whole period. In our experience, the preterm and underweight babies represent on average 11.3% of all the neonates. We screened 60,408 babies and recalled 196 of them, with a recall rate of 0.32%. Out of the 196 positive samples, 11 were true positive while one more positive (false negative case) was diagnosed following a metabolic imbalance of the baby, during her first months of life. Another two babies, giving a positive screening test, died after few days of life while the confirmatory DNA test was still pending. The negative predictive value (NPV) is 99.99% and the positive predictive value (PPV) is 6.12%, with an overall specificity of 99.70% and sensitivity of 92.31%, excluding the two cases not yet confirmed at the DNA level. The overall frequency of non-PKU metabolic disorders in the screening panel was 1:6041 newborns or 1:5034 when including newborns with an affected mother.

Our experience shows a prevalence of organic acidemias (7 cases out of 12, accounting for 58%, frequency of 1:8630), an equal incidence of amino acid disorders and mitochondrial fatty acid beta-oxidation defects (2 cases respectively, 16.7%, prevalence of 1:30,204), and only one case of urea cycle disorders (8.3%, prevalence of 1:60,408). Moreover, our data showed a singular prevalence of the disorders in female babies (75%) as, among the three male babies, two reflected the metabolic disorder of their mothers. Table 2 describes our clinical cases.

### 3.1. Amino Acid Disorders (AA)

Hypermethioninemia was diagnosed in a Caucasian preterm female baby which was recalled to perform plasma and urine amino acid profile. High and persistent levels of methionine in plasma and urine without homocystinuria supported the diagnosis of hypermethioninemia.

Non-ketotic hyperglycinemia was diagnosed in a Caucasian female baby who displayed seizures and alterations in brain development since her first weeks of life. She had shown an elevated glycine liquor to plasma ratio (0.20). Further MRI revealed hypogenesis of the corpus callosum, an abnormal gyrus, and the hypogenesis of the cerebellum. Electroencephalogram (EEG) showed suppression burst and hypsarrhythmia. The baby, 6 months old, showed all the typical signs of the disease such as hypotonia, apnea, lethargy, hiccups, as well as intractable myoclonic jerks/seizures, spasticity, and alterations in brain development.

### 3.2. Urea Cycle Disorders (UCD)

An argininosuccinic acid synthetase deficiency was identified in a female baby. The infant had shown lethargy, poor feeding, vomiting, and loss of consciousness since her first day of life. Hyperammonemia was also found. The baby was immediately admitted to our hospital in order to start the follow-up treatment.

### 3.3. Mitochondrial Fatty Acidbeta-Oxidation Defects (FAO)

A female infant with a positive screening result for C4 was recalled but no elevation of any acylcarnitines in her second DBS was found. The organic acid profile showed a very high amount of ethylmalonic acid. She is now one and a half years old and she is still asymptomatic.

A female baby with very high values of medium chain acylcarnitines was immediately admitted to our hospital to perform a confirmatory test. The new DBS analyses showed a decrease of the acylcarnitine values though they were still above the cut-off values. She is one year old and still shows lethargic episodes and feeding refusal.

### 3.4. Organic Acidemias (OA)

Diagnosis of MMA with homocystinuria was made in a preterm female infant. She was one of triplets. Clinical signs started to appear in the first month of life. She shows persistent excretion of methylmalonic acid in urine and further genetic investigations are in progress.

MMA was also diagnosed in a male baby with positive family history (one brother with MMA). He showed hypotonia, lethargy, and extrapyramidal symptoms during his first hours of life, so he was immediately admitted to our hospital and treated. Abnormal and persistent amounts of methylmalonic acid in the urine confirmed the diagnosis. Despite diet and therapy, he had a significant developmental delay.

Propionic acidemia was diagnosed in a Caucasian female baby which was recalled for a confirmatory test. She showed abnormal excretion of propionic acid in the urine. Typical clinical signs, such as poor feeding, vomiting, loss of appetite, hypotonia, and lethargy that appeared after the few days of life. The baby was immediately admitted to our hospital to start the follow-up treatment. She died when she was three years old.

One mother with a Vitamin B12 deficit was diagnosed because her firstborn male showed an elevated C3 value with low levels of methionine. Normalization of the C3 levels occurred in the retest specimen, although the urinary organic acid profile showed the excretion of methylmalonic acid that disappeared in the third specimen collected one week later. Low levels of B12 were found in the mother (116 pg/mL). Another mother with 3MCC deficit was diagnosed because her infant had a very high C5OH\C4DC value and low C0 value. The baby was recalled but the C5OH\C4DC value drastically decreased. Meanwhile, the free carnitine level had increased. The mother’s anamnesis disclosed a peculiar symptomatology for 3MCC deficiency, such as vomiting, weakness, muscular hypotonia, and metabolic crisis, following the introduction of a protein-rich diet. The 3MCC deficiency was confirmed by the presence of 3-OH-isovaleric acid and 3-methylcrotonylglicine in her urine.

A female baby showed a mild increase of C4OH\C3DC. She was recalled but a negative DBS analysis was obtained. However, she showed abnormal and persistent urinary excretion of malonic acid and manifested severe cardiomyopathy. She frequently has episodes of hypoglycemia.

The deficit of 3-OH-3MethylglutarylCoA lyase was the only false negative case in our experience. The female baby did not show a typical profile because the DBS displayed mildly elevated C6DC and C5OH\C4DC below the cut-off value. All secondary markers and ratios were normal. However, a few months later, the baby developed severe hypoglycemia during a fever. Urine collected during the hypoglycemic crisis showed 3-OH-3-methyl glutaric acid and 3-methyl glutaric acid disclosing the metabolic disease.

Two more metabolic diseases not yet molecularly confirmed were found in two babies who died a few days after birth. One female showed a profile compatible with glutaric aciduria type II, whereas a preterm male showed a typical pattern of LCHAD (long-chain 3-hydroxyacyl-CoA dehydrogenase deficiency)\MTP (mitochondrial trifunctional protein deficiency) deficit.

## 4. Discussion

The results of our seven-year experience using MS/MS show an overall specificity of 99.70% and sensitivity of 92.31%. The NPV is 99.99% and PPV 6.12%. The last annual report of the Italian Society for the Study of Metabolic Diseases and Neonatal Screening (SIMMESN) reports the incidence data for AA = 1:18,080, FAO = 1:4364, OA = 1:11,005. Our experience shows a lower incidence rate for AA and FAO (1:30,204) but a slightly higher incidence rate for OA (1:8630). Moreover, our data show a prevalence of the disorders in female babies (75%). These data highlight the importance of the NBS as a basic health program to avoid the underestimation of rare diseases, especially in a population which is the genetic result of many different groups. Arabs and Normans were the two major groups that have ruled Sicily in medieval times and their legacy still persists. Indeed, as it is known, organic acidemias are the most commonly diagnosed inborn errors of metabolism in different Asiatic countries [10,11,12] while mitochondrial fatty acid beta-oxidation defects seem to be the most common deficiency in North European population groups [13]. Based on our experience, we wish to stress that the diagnoses of malonic acidemia and 3 hydroxy-3 methyl glutaric acidemia deserve specific investigations even when there are not textbook alterations of the metabolic profiles. Indeed, since C3DC was included in the MS-MS screening panel of the NBS programs, new cases of MCD have been reported [14,15,16]. However, in some cases, a mild C3DC value from the NBS analysis was obtained [15] or the first acylcarnitine profile after NBS did not show C3DC elevation [16]. Moreover, our experience highlights how the normalization of C4 in a second specimen does not reassure the absence of the disease as further investigation is needed to evaluate the presence of urine organic acids.

## 5. Conclusions

To improve our results, a primary goal is the development of a second-tier test with the inclusion of more specific markers in order to decrease the false positive rate and to improve the PPV. Indeed, many easy second tier strategies are now available to reduce false tests [17,18,19,20]. The inclusion of ADA-SCID (adenosine deaminase-severe combined immunodeficiency) in expanded newborn screening by tandem mass spectrometry [21], as well as the expansion of the NBS programs to include lysosomal storage disorders (LSDS), could be the second target to achieve. Newborn screening for LSDs has become a growing area of debate because of the availability of treatments that might produce an optimal clinical outcome when started very early in life [22,23,24].

## Figures and Tables

**Table 1:** The number of screened babies (as a sum of full-term and preterm babies), preterm babies, total analysis, and recall performed during the seven years.

Year	Screened Babies	Preterm Babies	Total Analysis	Recall
2011	1722	175	2052	2
2012	7315	740	9422	26
2013	8675	935	9941	32
2014	10,141	1050	11,377	28
2015	11,463	1323	13,169	40
2016	10,746	1343	12,384	19
2017	10,346	1244	11,969	49

**Table 2:** The clinical cases diagnosed by newborn screening using tandem mass spectrometry.

Class	Deficit (Acronym)	Marker Concentration * (Cut off)	Gene	Mutation
AA	Hypermethioninemia (MET)	Met = 207.0 (31.0)	*MAT I*, *MAT III*	in progress
AA	Non ketotic hyperglycinemia (NKG)	Gly = 1790 (740)	*GLDC* *AMT*	in progress
UCD	Argininosuccinic acid synthetase deficiency (CIT I)	Cit = 1412.8 (28.8)	*ASS1*	compound Heterozygous c.323G > T exon 5 c.1022C > T exon 14
FAO	Short chain acylCoA dehydrogenase deficiency (SCAD)	C4 = 1.06 (0.55)	*ACADS*	Homozygous c.625G > A
				Heterozygous c.301G > A
FAO	Medium chain acylCoA dehydrogenase deficiency (MCAD)	C6 = 2.46 (0.10)	*ACADM*	Homozygous c.253G > C
		C8 = 20.82 (0.12)		
		C10 = 1.68 (0.18)		
		C10:1 = 0.33 (0.10)		
		C8/C10 = 12.39		
OA	Methylmalonic acidemia (MMA) with homocystinuria	C3 = 11.60 (3.84)	*MMACHC*	Heterozygous c.271-272dupA
OA	Methylmalonic acidemia (MMA) with homocystinuria	C3 = 49.79 (3.84)	*MMACHC*	**
OA	Propionic acidemia (PA)	C3 = 7.99 (3.84)	*PCCA*	Homozygous c.1899_1953del
OA	Maternal Vitamin B12 deficiency	C3 = 7.15 (3.84)	*GIF*	n.a.
OA	Maternal 3-methyl crotonylCoA carboxylase deficiency (3MCC)	C5OH\C4DC = 4.68 (0.32)	*MCCC1* *MCCC2*	n.a.
		C0 = 4.69 (8.00)		
OA	MalonylCoA decarboxilase deficiency	C4OH\C3DC = 0.41 (0.25)	*MLYCD*	Homozygous c.1283C > T
OA	3-OH-3-methylglutarylCoA lyase	C6DC = 0.72 (0.36) C5OH\C4DC = 0.26 (0.32)	*HMGCL*	n.a.

AA = amino acid disorders, OA = organic aciduria, FAO = fatty acid oxidation deficiency; Met = methionine, Gly = glycine, Cit = citrulline, C0 = free carnitine, C3 = propionylcarnitine, C3DC = malonylcarnitine, C4 = butyrylcarnitine, C4DC = methylmalonylcarnitine, C4OH = 3-hydroxybutyrylcarnitine, C5OH = 3-hydroxy -isovalerilcarnitine, C6 = hexanoylcarnitine, C6DC = adipylcarntine, C8 = octanoylcarnitine, C10 = decanoylcarnitine, C10:1 = decenoylcarnitine; * unit of measurement for concentrations μM; ** genetic analysis refused by parents; n.a. = not available.
